# Supplementary material for: Metabolic model predictions enable targeted microbiome manipulation through precision prebiotics
Source: Microbiol Spectr. 2024 Jan 17;12(2):e01144-23. doi: 10.1128/spectrum.01144-23 (PMC10846184; doi:10.1128/spectrum.01144-23)
Supplement: Supplementary figures — Figures S1 and S2. [file spectrum.01144-23-s0001.docx]

# Supplementary Figures
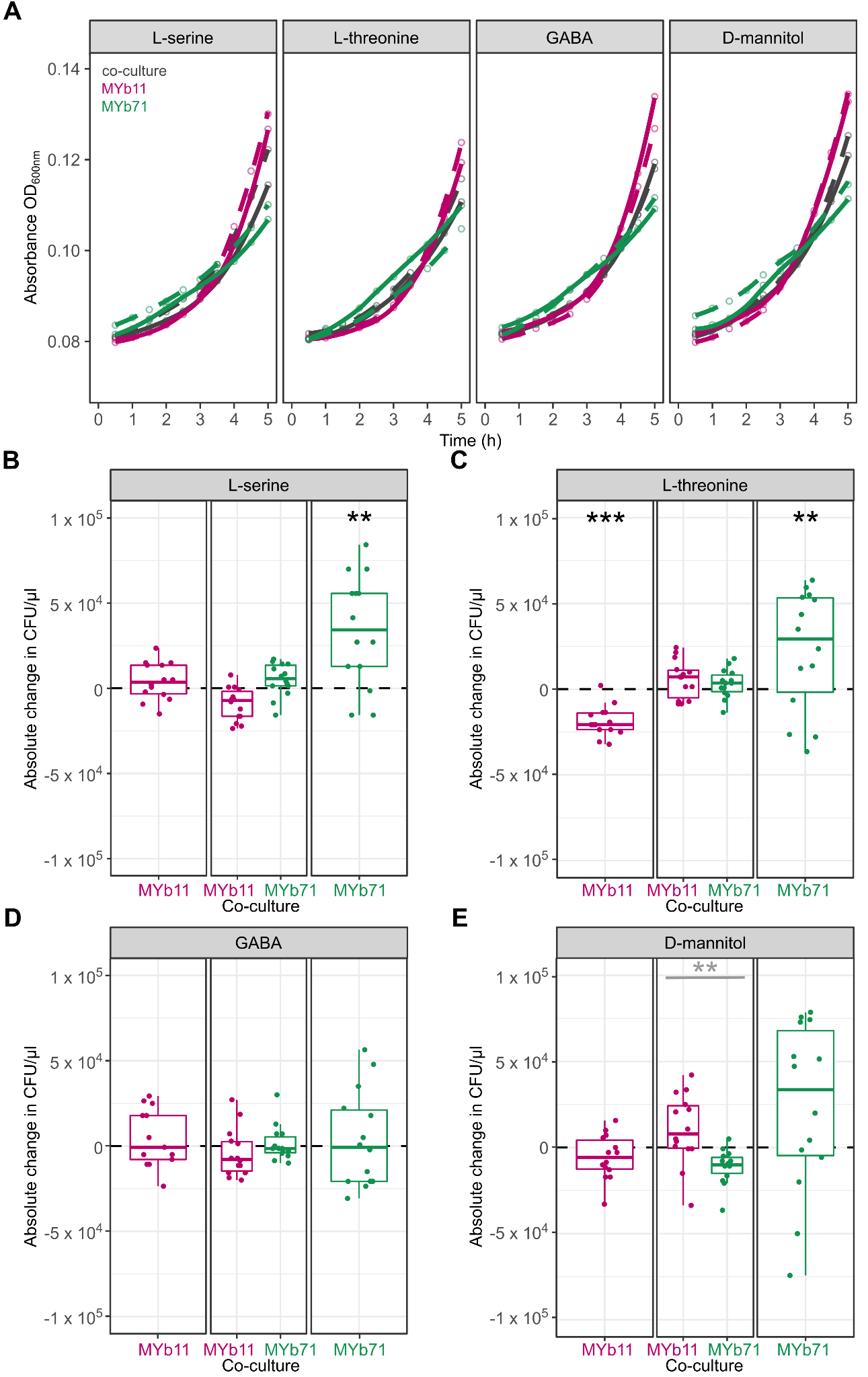


**Supplementary Figure S1: *In vitro* growth of *Pseudomonas lurida* MYb11 and *Ochrobactrum vermis* MYb71 in mono- and co-culture in the presence and absence of four different supplements for 5** **h**. **(A)** Growth curves of mono- and co-cultures (gray) of MYb11 (pink) and MYb71 (green) in liquid NGM for 5 h either with 10 mM of L-serine, L-threonine, GABA, and D-mannitol (solid line) or without supplementation (dashed line). **(B, C ,D, E)** Colony-forming units (CFU/µl) in mono-cultures of MYb11 (left) and MYb71(right) or in co-culture (middle) after 5 h of 10 mM of the respective supplement. Shown are boxplots with the median as a thick horizontal line, the interquartile range as box, the whiskers as vertical lines, and each replicate depicted by a dot. Every replicate was normalized by subtracting the non-supplemented median (dashed line) of the respective bacteria. Statistical differences were determined by Wilcoxon signed rank test and are indicated by asterisks (*** *p* < 0.001, ** *p* < 0.005, * *p* < 0.05). Black asterisks indicate statistical comparisons between supplemented and non-supplemented median, gray asterisks indicate statistical comparisons between supplemented medians of MYb11 and MYb71. n = 5-14.


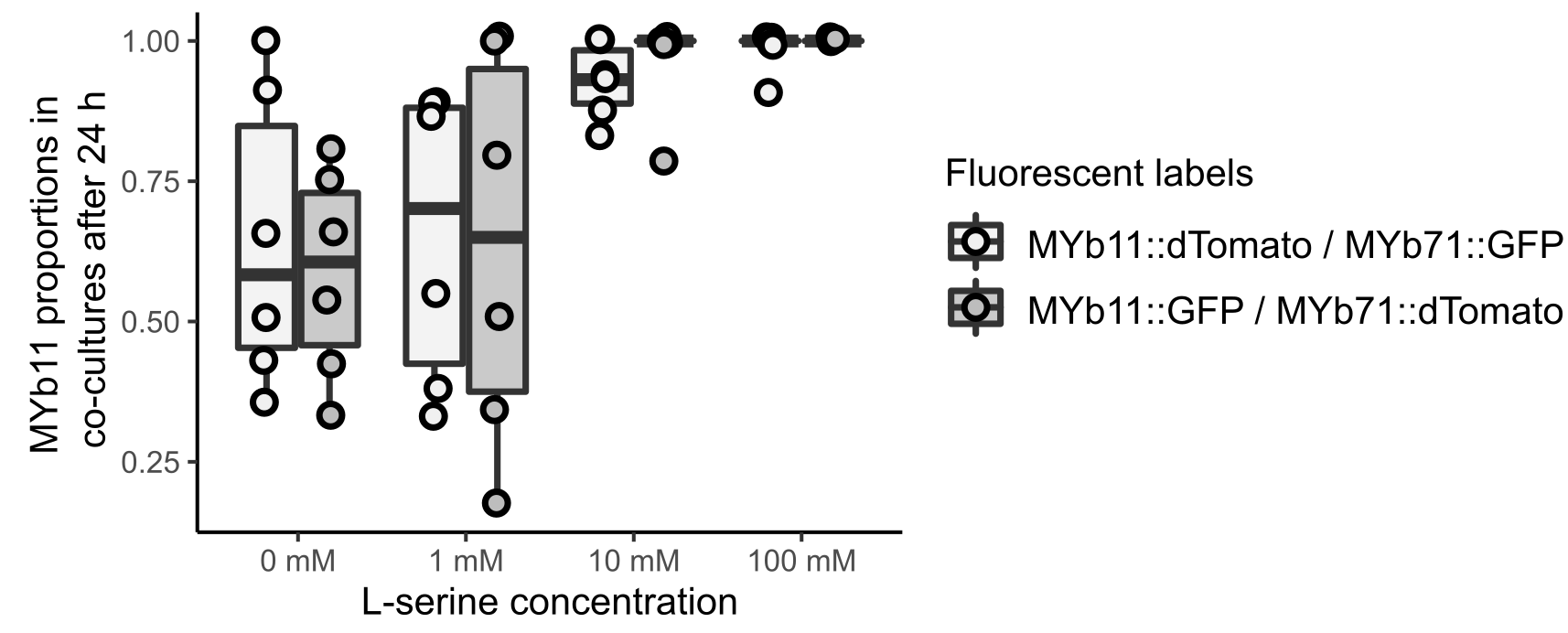


**Supplementary Figure S2. No difference in relative growth rates of differentially labeled bacterial strains.** Colony forming units of co-cultures of either MYb11::dTomato/MYb71::sfGFP or MYb11::GFP/MYb71::dTomato were quantified after 24 h of growth in liquid NGM. There was no difference between the two fluorescent labeling systems at any concentration of L-serine (GLM, *p* = 0.528 for main effect of fluorescence; and *p* = 0.379 for interaction between fluorescence and concentration). (See also: Supplementary Table S20)
